# Sleep deprivation increases levels of the synaptic density marker SV2A in the human brain

**DOI:** 10.1371/journal.pbio.3003816

**Published:** 2026-06-23

**Authors:** David Elmenhorst, Anna L. Foerges, Ali Gordji-Nejad, Eva-Maria Elmenhorst, Tina Kroll, Andreas Matusch, Simone Beer, Bernd Neumaier, Philipp Krapf, Christoph Lerche, Alexander Drzezga, Andreas Bauer

**Affiliations:** 1 Institute of Neuroscience and Medicine (INM-2), Forschungszentrum Jülich, Jülich, Germany; 2 Department of Nuclear Medicine, University of Cologne, University Hospital Cologne, Cologne, Germany; 3 Institute of Aerospace Medicine, German Aerospace Center (DLR), Cologne, Germany; 4 Medical Faculty, Institute of Occupational, Social, and Environmental Medicine, RWTH Aachen University, Aachen, Germany; 5 Institute of Neuroscience and Medicine (INM-5), Forschungszentrum Jülich, Jülich, Germany; 6 Institute of Radiochemistry and Experimental Molecular Imaging (IREMB), University Hospital Cologne, Germany; 7 Institute of Neuroscience and Medicine (INM-4), Forschungszentrum Jülich, Jülich, Germany; 8 Clinical Research, German Center for Neurodegenerative Diseases (DZNE), Bonn-Cologne, Germany‌‌; 9 Medical Faculty, Department of Neurology, Heinrich-Heine-University Düsseldorf, Düsseldorf, Germany; Columbia University Irving Medical Center, UNITED STATES OF AMERICA

## Abstract

Sleep is essential for synaptic homeostasis, a proposed mechanism whereby wakefulness leads to synaptic potentiation and sleep facilitates synaptic down-selection. Synaptic vesicle glycoprotein 2A (SV2A), whose availability is quantifiable by [¹⁸F]SynVesT-1 positron emission tomography (PET), is commonly interpreted as a proxy for synaptic density. In this randomized study, we examined 40 healthy adults (mean age 27.5 ± 6.5 years) who underwent two [¹⁸F]SynVesT-1 PET scans on consecutive days. Half of the participants were assigned to the normal sleep (i.e., control) condition and half to the sleep deprivation condition. Scans were performed at the same circadian time point, approximately 4 h after awakening in the control group and during baseline in the sleep deprivation group or after ~28 h of continuous wakefulness in the sleep deprivation group after sleep deprivation. Sleep deprivation led to significant increases in synaptic vesicle glycoprotein 2A binding in multiple brain regions, including the thalamus (+4.6%), hippocampus (+5.6%), and parietal cortex (+3.2%), whereas no changes were observed in controls. The degree of increase in synaptic vesicle glycoprotein 2A positively correlated with elevated slow wave activity during recovery sleep, a physiological marker of sleep pressure. These findings provide in vivo support for the synaptic homeostasis hypothesis in humans and suggest that synaptic vesicle glycoprotein 2A PET imaging is sensitive to sleep-wake dependent synaptic plasticity.

**Trial Registration:**

The study was prospectively registered on 19.01.2022 here: German Clinical Trials Registry: DRKS # DRKS00027867, https://drks.de/search/en/trial/DRKS00027867.

## Abstract 2

The synaptic homeostasis hypothesis proposes that wakefulness increases synaptic strength, while sleep facilitates synaptic down-selection, but direct evidence for this phenomenon in the human brain remains scarce. This PET imaging study provides new evidence that sleep deprivation increases synaptic density across the human brain.

## Introduction

The synaptic homeostasis hypothesis (SHY) [1–4] posits that wakefulness promotes synaptic potentiation due to environmental interactions and learning [5]. The strengthening of connections during waking elevates energy consumption, results in the accumulation of proteins and receptors that compete for the limited anatomical space in the skull and diminishes the signal-to-noise ratios in the neuronal network, ultimately saturating the capacity for learning. Sleep allows for synaptic down-selection, preserving energy and network efficiency. While the SHY has been supported by anatomical and molecular studies in animals, human evidence has remained limited due to the invasive nature of most techniques for quantifying synaptic strength.

Studies in animals indicate that anatomical or molecular markers of synaptic strength increase during wake and decline during sleep [6]. Firing rates in rodents indicate increased cortical excitability during wakefulness and decreased cortical excitability during sleep. In humans, cortical excitability is an indirect measure of plasticity. Findings from studies using transcranial magnetic stimulation (TMS) translated the findings from the above-mentioned rodent studies (reviewed in [7]). However, some in-vitro and in-vivo studies of synaptic strength in animals reveal opposite results, which may be due to differences in the type of marker, examined brain regions, cortical layers, or housing of animals (reviewed in [8]).

Synaptic vesicle glycoprotein 2A (SV2A) [9] is an integral membrane protein located on synaptic vesicles. Recent advances in PET imaging with tracers such as [¹⁸F]SynVesT-1 enable the noninvasive measurement of SV2A binding in the living human brain [10,11], allowing new opportunities to examine state-dependent synaptic changes. However, whether this reflects presynaptic terminal number, vesicle complement, SV2A expression per vesicle, or excitatory/inhibitory-synapse composition cannot be resolved with in vivo imaging. While SV2A availability is commonly interpreted as a proxy measure of synaptic density, we refer to it here as ‘SV2A-indexed synaptic density’ to reflect this interpretation while acknowledging its underlying biological ambiguity.

Homeostatic sleep pressure, quantified as slow wave activity (SWA), a physiological indicator of sleep need, likewise increases with time awake and declines during sleep (two-process model of sleep-wake regulation [12]).

**Fig 1:**
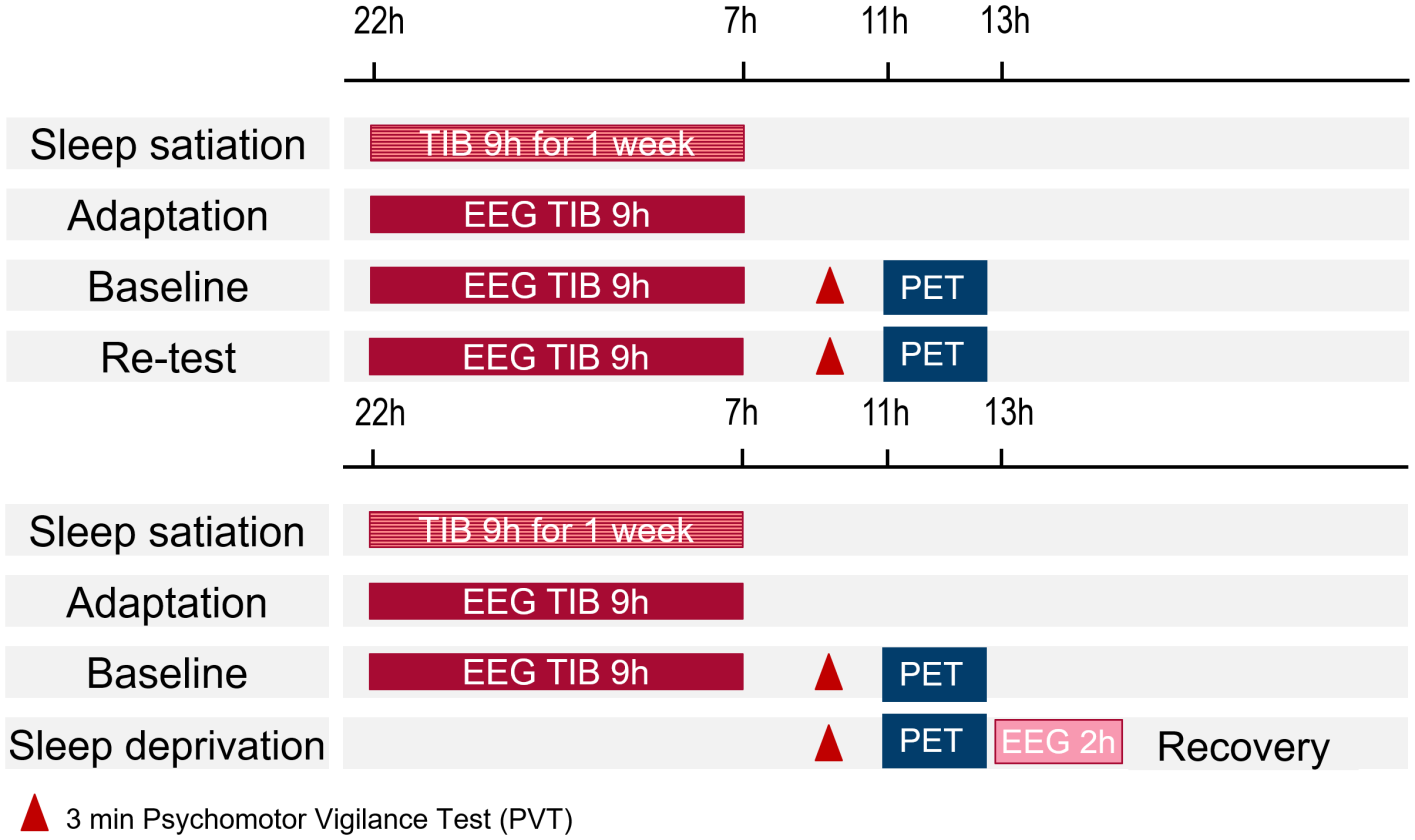
Study design (TIB time in bed).

The aims of this study were to evaluate in healthy volunteers with two consecutive SV2A PET scans: (*i*) to what extent SV2A-indexed synaptic density is increased after 28 h of sleep deprivation compared to a baseline condition and to data from a rested test-retest control group (primary outcome); and (*ii*) whether the buildup of sleep pressure after sleep deprivation compared, with preceding baseline sleep, is correlated with a higher SV2A-indexed synaptic density (exploratory analysis). Here we report results from a cohort (*n* = 40, f/m 14/26) which was randomized prior to inclusion to either normal sleep (control group, *n* = 20) or acute total sleep deprivation (sleep deprivation group, *n* = 20) -between scans (Fig 1).

## Results

### Demographic and scanning characteristics

The control and the sleep deprivation groups did not differ in demographic and scanning characteristics (S1 Table), but, as intended, differed in the amount of time spent awake before the start of the second scan.

### SV2A-indexed synaptic density increases after sleep deprivation

**Fig 2:**
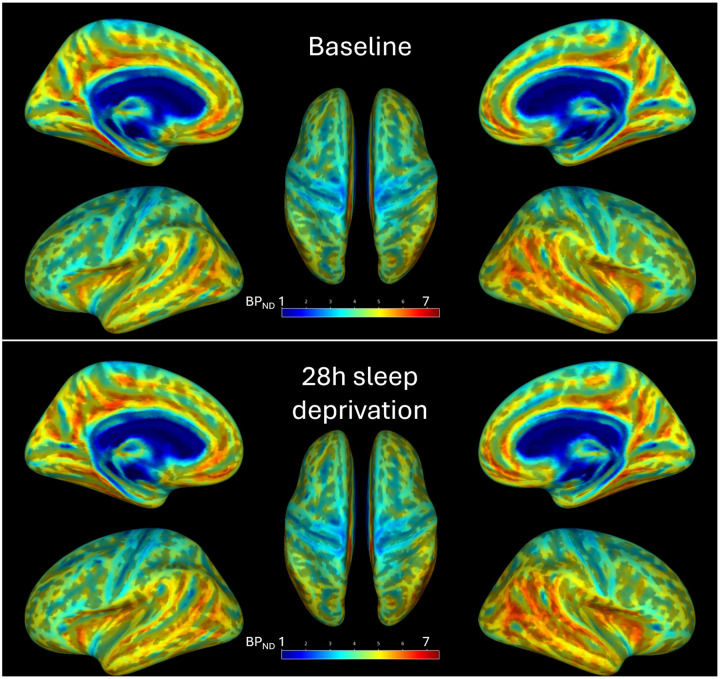
Inflated volume-mapped parametric images of cortical SV2A-indexed synaptic density (SV2A binding potential BP_ND_, sleep deprivation group, *n* = 20) overlayed on the FsAveragesurface as implemented in the CAT12 toolbox.

**Fig 3:**
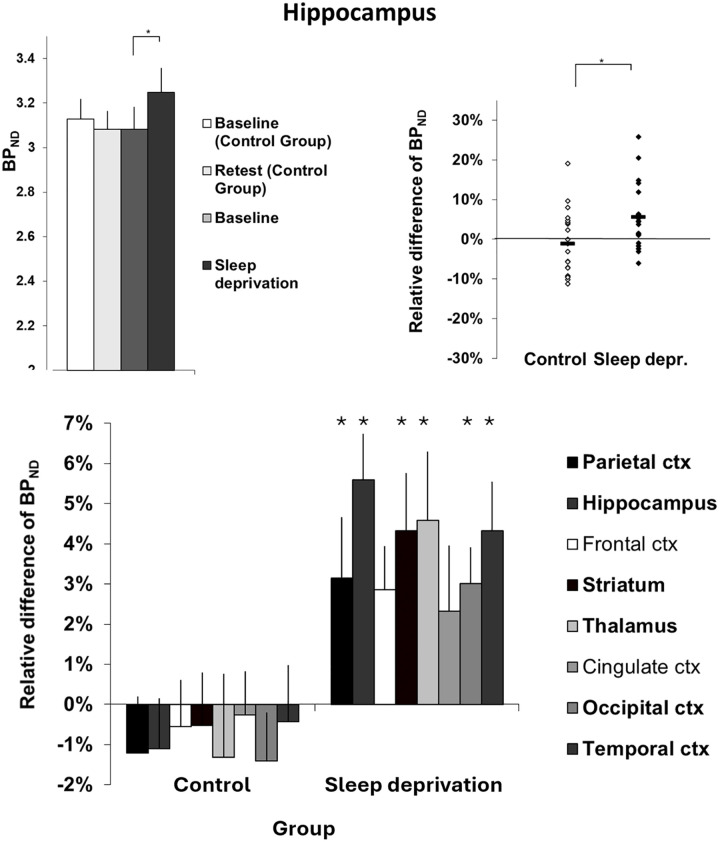
Upper panel: Average absolute values (upper right) and individual relative differences [(scan2 – scan1)/scan1] of SV2A-indexed synaptic density (SV2A binding potential BP_ND_) in hippocampus. Lower panel: Regional average relative differences. Error bars indicate SEMs. The asterisks represent significant differences between groups in the mixed model (group: *p* = 0.0103, region: *p* = 0.34, interaction *p* = 0.43, two groups *n* = 20, eight regions, post-hoc mult. comp. correction: false discovery rate). The data underlying this Figure are available here: S1 Data.

We calculated random-subject mixed ANOVAs on the difference between both scans with group (control and sleep deprivation), brain region (*n* = 8) and the interaction between group and brain region as fixed factors. False discovery rate adjusted post-hoc comparisons revealed that SV2A-indexed synaptic density was increased after sleep deprivation (Figs 2 and 3).

The sleep deprivation group exhibited significant increases in SV2A binding potential in six of eight brain regions analyzed. For example, relative increases were observed in the thalamus (+4.6 ± 7.3%, *p* = 0.039), hippocampus (+5.6 ± 8.2%, *p* = 0.0195), and parietal cortex (+3.2 ± 5.1%, *p* = 0.022). In contrast, no significant differences were observed in the control group (Δ range: −0.3% to −1.4%, all p > 0.2), confirming the test-retest stability of SV2A PET measurements. Intraclass correlation coefficients (ICCs) ranged from 0.87 to 0.93 across regions in the control group.

Regional SV2A-indexed synaptic density values and statistics are presented in Table 1.

**Table 1:** Regional SV2A-indexed synaptic density values (binding potential BP_ND_).

BP_ND_	Scan	Group		Mixed model
Region		Control	Sleep Deprivation	p-value post-hoc mult. comp. correction: false discovery rate
Cingulate ctx	1	4.63 ± 0.4	4.59 ± 0.4	
	2	4.61 ± 0.3	4.7 ± 0.4	
	Δ	−0.27%	2.32%	0.081
Frontal ctx	1	3.98 ± 0.3	4.02 ± 0.4	
	2	3.95 ± 0.3	4.13 ± 0.4	
	Δ	−0.54%	2.85%	0.063
Hippocampus	1	3.13 ± 0.4	3.08 ± 0.3	
	2	3.08 ± 0.4	3.25 ± 0.4	
	Δ	−1.10%	5.60%	**0.0195**
Occipital ctx	1	4.35 ± 0.4	4.34 ± 0.5	
	2	4.28 ± 0.4	4.47 ± 0.5	
	Δ	−1.40%	3.02%	**0.0195**
Parietal ctx	1	4.17 ± 0.4	4.16 ± 0.4	
	2	4.11 ± 0.3	4.29 ± 0.5	
	Δ	−1.21%	3.15%	**0.022**
Striatum	1	3.76 ± 0.3	3.71 ± 0.4	
	2	3.74 ± 0.4	3.86 ± 0.5	
	Δ	−0.52%	4.33%	**0.024**
Temporal ctx	1	4.18 ± 0.4	4.13 ± 0.4	
	2	4.15 ± 0.4	4.3 ± 0.4	
	Δ	−0.43%	4.33%	**0.0195**
Thalamus	1	2.72 ± 0.3	2.61 ± 0.3	
	2	2.68 ± 0.3	2.73 ± 0.3	
	Δ	−1.31%	4.59%	**0.039**

Mean ± SD, significant differences between groups are marked in bold. The data underlying this Table are available here: S1 Data.

### The increases in SV2A-indexed synaptic density and SWA after sleep deprivation are correlated

**Fig 4:**
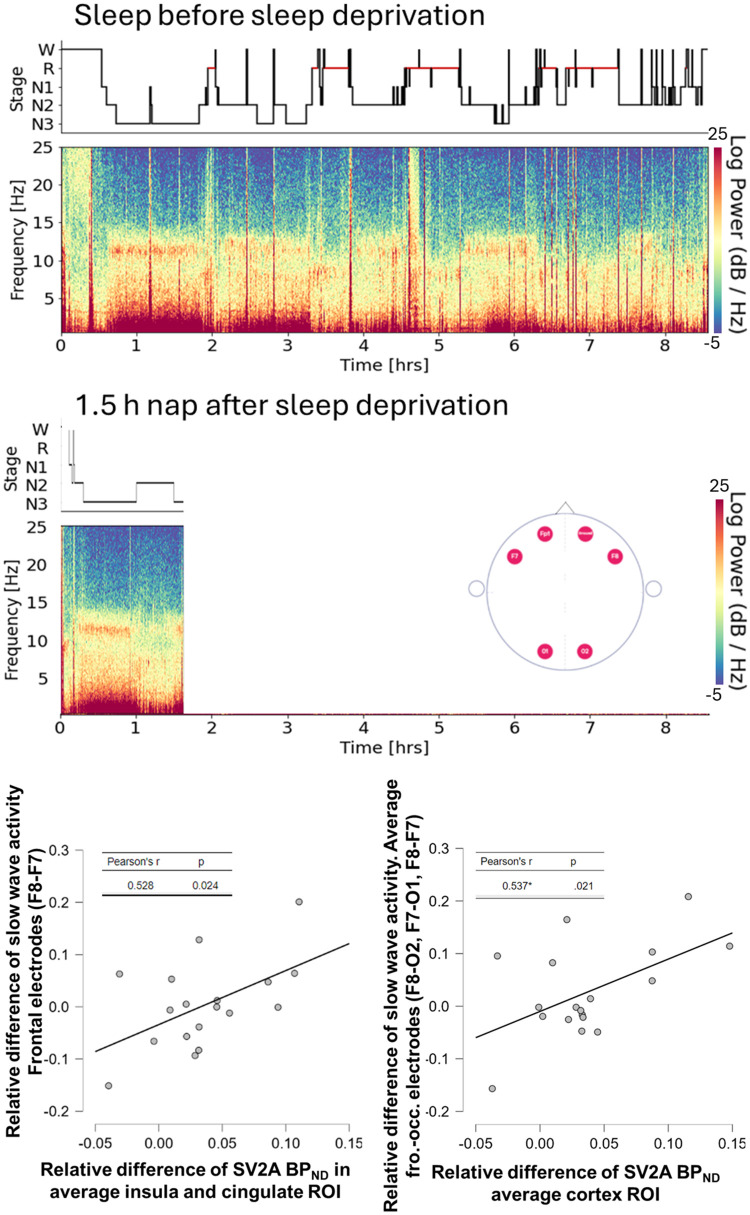
Sleep hypnogram staging and single channel multitaper spectrograms of a representative subject. The upper panel shows baseline sleep and the middle panel the recovery nap after sleep deprivation. A small insert in the middle panel depicts the electrode positions. The relative difference of slow wave activity (SWA) during the first 1.5 h of both recordings was used to explore the relationship between the buildup of sleep propensity and the increase of SV2A-indexed synaptic density in the lower panel (relative differences of sleep EEG SWA vs. relative change in SV2A-indexed synaptic density (scan2-scan1)/scan1). The data underlying this Figure are available here: S3 Data.

Sleep-deprived participants had a 2-h recovery sleep opportunity after the second PET scan. SWA from sleep-EEG was calculated for a duration of 1.5 h after sleep onset during baseline and recovery sleep. Cognitive performance was measured with a psychomotor vigilance task (PVT) and sleepiness was assessed with the Karolinska Sleepiness Scale (KSS) right before the PET scans. The increase in SV2A-indexed synaptic density after sleep deprivation correlated with the increase in SWA during recovery sleep, both compared to baseline (S2 Table). Fig 4 shows scatter plots of the main generator regions of SWA (cingulate and insula according to Murphy and colleagues 2009 [13]).

Such correlations were not significant for cognitive performance and sleepiness. Nevertheless, sleep deprivation led to significantly increased sleepiness and impaired cognitive performance (S3 Table).

## Discussion

Our findings provide in vivo evidence that extended wakefulness increases SV2A binding in the human brain, consistent with a synaptic potentiation during wake. These changes were regionally widespread and correlated with increased SWA during recovery sleep, supporting the synaptic homeostasis hypothesis.

Interestingly, the observed increases in SV2A were modest (~2%–6%), yet comparable to structural changes reported in animal studies after sleep deprivation. These relatively small but reliable effects are notable given the temporal offset (~4.2 h post-wake) before baseline PET scanning, which may have allowed partial early wake‑dependent changes of synaptic components to develop, diminishing the magnitude of the difference compared with the sleep‑deprived condition.

The magnitude of increase that we observed lies between findings from animal experiments that used in vivo methods of spine counting by microscopy or that collected brain tissue samples immediately before lights off/on or after extended wake periods. Using longitudinal two-photon imaging of yellow fluorescent protein expressing apical dendrites of layer V pyramidal neurons in mice, the dynamics of spine turnover revealed a net increase of about 3% after 6h of extended wakefulness [14]. Block-face scanning electron microscopy on mouse brain primary somatosensory and motor cortices indicated that the dendritic axon spine interface decreases by about 18% during sleep compared to spontaneous wake and enforced wakefulness [15]. Using homogenates of cortex preparations in mice and Western Blot for the AMPA receptor subunit GluA1 showed about a 40% higher amount after wakefulness compared to sleep [16]. Our subjects were scanned a few hours after awakening, which might have attenuated the observed difference to the sleep deprivation effect. However, direct comparison of effect magnitudes across species should be interpreted with caution. Differences in brain architecture, sleep-wake patterns, tracer biology, and the spatial scales captured by PET versus microscopy or biochemical assays limit the extent to which quantitative values can be directly equated. Thus, our comparison is intended to provide qualitative context rather than imply strict numerical equivalence.

SV2A binding has been shown to correlate with synaptophysin in postmortem tissue [17–19], an integral synaptic vesicle membrane protein that interacts with the SNARE complex and regulates vesicle formation and neurotransmitter release. Studies in male rodents have shown no change or more often decreases in synaptophysin after 24–96 h of sleep deprivation using cage shaking or mainly the multiple platform over water method, where animals will fall into the water during REM sleep associated muscle atonia [20–23] (reviewed in [24]). However, the translation of results from rodents to humans is limited due to the methodological differences used to enforce wakefulness. Most rodent experiments rely on long and often stressful (life-threatening) deprivation protocols, which induce physiological stress responses that are difficult to separate from sleep loss effects.

The observed association between SWA and SV2A-indexed synaptic density is in line with previous findings: Chemogenetically, it has been shown that local dendritic spine enlargement and synaptic potentiation in the prefrontal cortex of mice induces longer and deeper sleep (increasing local SWA) [25]. Slow wave sleep drives reduction of synaptic AMPA receptor levels in rat neocortex and hypothalamus [26]. The glutamatergic synapse is the most abundant type, but to date there is no data available that quantified the impact of sleep or sleep deprivation on AMPA receptors in humans yet. Using PET in humans, increased metabotropic glutamate receptor (mGluR5) densities after sleep deprivation were shown to be positively correlated with the rebound in SWA in NREM sleep after prolonged wakefulness [27]. Similarly, recovery sleep after sleep deprivation decreased the elevated [28] adenosine A_1_ receptor availability and correlated negatively with the homeostatic sleep pressure measured as amount of deep sleep (N3) in the first sleep cycle [29]. Other neuroreceptors which showed an increase after sleep deprivation were serotonin 2a receptors in which, however, an association with the buildup of sleep pressure during recovery sleep was not investigated [30].

Our findings align with multimodal evidence from magnetic resonance spectroscopy (MRS) studies showing that sleep deprivation increases neurochemical markers associated with synaptic activity. Converging proton MRS evidence demonstrates that prolonged wakefulness elevates glutamatergic tone—reflected in increased glutamate or GLX levels, higher Glu/GABA ratios, and corresponding increases in mGluR5 availability—and complementary MRS findings from multimodal PET/MRS work further show sleep-loss–related changes in metabolites such as myo-inositol and glycine, which are linked to astroglial regulation of glutamatergic signaling and sleep pressure [27,31,32].

SV2A binding did not correlate with behavioral changes in psychomotor vigilance or subjective sleepiness. This dissociation suggests that molecular indices of synaptic plasticity may operate independently from overt behavioral impairments.

The interpretation of an increased SV2A PET signal after sleep deprivation must be approached with appropriate caution. SV2A is a vesicular glycoprotein found on synaptic terminals and is commonly used as a proxy for synaptic density. However, it is important to note that this marker reflects synaptic vesicle glycoprotein abundance and does not directly measure synaptic efficacy, functional plasticity, or postsynaptic changes. It is estimated that each vesicle contains roughly five SV2 molecules [33], and whether this number is fixed or variable under different physiological conditions such as sleep deprivation is currently unknown. Given that SV2A is present in both excitatory and inhibitory synapses, the observed fluctuations in tracer binding may reflect a net change in the brain’s excitatory/inhibitory (E/I) tone following sleep loss. The inherent lack of specificity for a particular neurotransmitter system remains a limitation in interpreting whether these results represent a targeted or global synaptic response.

Comparisons can be made to in-vitro techniques that determine synaptic density, such as spine counting. Potentiation or depotentiation at a synapse leads to linked changes in anatomical/molecular proxies of synaptic strength (reviewed in [34]): spine head or bouton volume correlates with the area of the post synaptic density and the number of presynaptic vesicles in rats [35,36] and humans [37]. The advantage of our approach is a whole-brain assessment of SV2A-indexed synaptic density applied longitudinally in humans.

Moreover, the study employed a modest sample size (*n* = 20 per group), consistent with the logistical and financial constraints of PET imaging but still limited in its ability to detect smaller effect sizes or subgroup effects, such as potential sex-based differences in synaptic plasticity. Exploratory analyses did not reveal statistically significant sex effects, though females showed numerically greater SV2A increases in several regions, warranting follow-up in larger cohorts. The sample size is though larger than what has been published from microscopic counting of synapses at dendrites of usual very few animals.

Changes in synaptic density may be inferred from SV2A binding only under the assumption that the number of vesicles per terminal or the number of active synapses has increased. It remains unclear whether such changes reflect new synapse formation, vesicle accumulation, or increased vesicle turnover. Nevertheless, prior studies have demonstrated that SV2A binding is stable over short timescales and is not acutely modulated by neural activity, as shown by PET-fMRI experiments where robust BOLD activations failed to influence SV2A binding in humans during visual stimulation [38].

Another critical consideration concerns PET quantification. The simplified reference tissue model 2 (SRTM2) was used with a population-based fixed k2’ value and an eroded centrum semiovale as the reference region. Although the centrum semiovale is commonly used in both [¹⁸F]SynVesT-1 and [¹¹C]UCB-J studies and shows high test-retest reproducibility [39,40], there is some evidence of low-level displaceable binding in this region, which could introduce systematic underestimation of the binding potential (BP_ND_). Despite this, our control group showed excellent test-retest reliability, with ICCs ranging from 0.87 to 0.93 and minimal relative differences (−0.3% to −1.4%), supporting the robustness of our approach.

Finally, although we observed no significant correlations between SV2A increases and behavioral measures such as psychomotor vigilance or subjective sleepiness, it is possible that molecular indicators of synaptic change are more sensitive or precede behavioral manifestations. Alternatively, behavioral measures may plateau or vary inter-individually, limiting their sensitivity to state-dependent synaptic dynamics.

It has been proposed that the short-lasting therapeutic success of sleep deprivation in some patients with depressive disorder can be explained by the increase in this homeostatic sleep drive [41]. The synaptic plasticity model of therapeutic sleep deprivation in major depression [42] combines the synaptic homeostasis hypothesis and the synaptic plasticity hypothesis of depression [43], suggesting that the strengthening of synapses by therapeutic sleep deprivation pushes the flawed long-term potentiation in these patients into a more advantageous range of associative plasticity. Since sleep deprivation as well as ketamine and electroconvulsive therapy are fast-acting strategies in the therapy of depression it can be hypothesized that these treatments may share a common pathophysiological mechanism. It has been shown that SV2A measured with PET is reduced in depressed patients with moderate-to-severe symptoms [44]. Electroconvulsive therapy increased SV2A-indexed synaptic density in a subgroup of patients who achieved remission [45] and likewise ketamine increased SV2A-indexed synaptic density in patients with a prior synaptic deficit [46].

While acute sleep deprivation may transiently up-regulate SV2A-indexed synaptic density, future work should examine the synaptic consequences of repeated or chronic sleep loss. Chronic sleep restriction has been shown to have different adenosinergic dynamics compared with acute sleep deprivation [47] and which is associated with cognitive impairment [48], neuroinflammation, and accelerated neurodegeneration in both healthy and clinical populations. Longitudinal SV2A PET studies spanning recovery sleep and chronic sleep restriction protocols will be essential to determine whether these synaptic changes are fully reversible or carry cumulative neural costs.

## Conclusions

This study provides direct molecular evidence that acute sleep deprivation increases SV2A binding in the human brain and that these changes are associated with elevated sleep pressure, as indexed by SWA. These findings support the synaptic homeostasis model in humans and highlight the utility of SV2A PET as a noninvasive tool for probing dynamic synaptic changes related to sleep-wake states. Given that synaptic deficits have been observed in major depression, and that both sleep deprivation and other fast-acting antidepressants (e.g., ketamine, ECT) increase SV2A binding, our findings suggest a potential shared neurobiological mechanism linking synaptic plasticity, sleep, and mood regulation.

## Materials and methods

All procedures were approved by the Ethics Committee of the regional Medical Board (Ärztekammer Nordrhein, Ethics committee number: 2019275) and the German Federal Office for Radiation Protection and carried out in accordance with the Declaration of Helsinki. Before participating in this study, participants gave written informed consent. The study was prospectively registered on 19.01.2022 with the German Clinical Trials Registry to provide transparency regarding objectives and methodology (DRKS # DRKS00027867, https://drks.de/search/en/trial/DRKS00027867), although it was not a clinical trial.

### Participants

Forty healthy human volunteers (14f; mean age 27.5 ± 6.5 (SD) years, range 20–45 years) participated in this study. Exclusion criteria were chronic neurological or psychiatric disorders, head trauma, sleep disorder, alcohol and illicit drug use, smoking, pregnant or breast-feeding females, current medication (except contraceptives) and an estimated habitual caffeine consumption above 500 mg/day. We checked for Zolpidem, Propoxyphene, Cotinine, Amphetamine, Methamphetamine, Morphine, Methadone, Phencyclidine, Nortriptyline, Tetrahydrocannabinol, Secobarbital, Oxazepam, Benzoylecgonine (Cocaine), and human chorionic gonadotropin with urinary tests.

### Protocol

The study consisted of a one-week ambulatory sleep satiation protocol (9 h time-in-bed (TIB) confirmed by actigraphy) with caffeine abstinence at home followed by an adaptation and baseline night in which sleep EEG was recorded. Sleep times were chosen to approximate individual habitual sleep times, i.e., either from 22:00 h to 07:00 h or from 23:00 h to 08:00 h. Following this ambulatory week, participants arrived in the laboratory in the morning for a set of measurements: a baseline psychomotor and sleepiness testing, and PET measurement. Two participants were studied at a time, with the second participant undergoing all procedures about 1 h after the first one. For better readability, the times for the first participant are given below.

Control participants were permitted to leave the laboratory after the first set of measurements while wearing an actigraph on their nondominant wrist. Upon their return the next day, actigraphy data were reviewed to rule out any daytime napping and a second set of the above-mentioned measurements followed. Sleep-deprived participants remained awake in the laboratory between these two sets of measurements. During this period, they were allowed to engage in nonstrenuous activities, such as conversing, watching videos, playing calm games, reading, or browsing the internet. Constant supervision by at least one study staff member ensured compliance. To maintain wakefulness, participants were not allowed to close their eyes longer than a blink; if they did so, the experimenter promptly intervened and encouraged them to stay awake.

Sleep-deprived participants had a 2-h recovery sleep opportunity (RS; 2 h TIB) in the sleep laboratory during which EEG was recorded in the afternoon after the second PET scan. Due to early awakenings or difficulties in falling asleep in some subjects, 1.5 h was the maximum sleep duration that could be analyzed uniformly across the group. Performance was assessed with a psychomotor vigilance test (PVT) before PET scans and at 03:00 h (sleep deprivation group only) to probe attention network functions.

### [^18^F]SynVesT1 PET data acquisition and analysis

[^18^F]SynVesT-1 was formulated and synthesized as follows: The fully GMP-compliant automated radiosynthesis of [^18^F]SynVesT-1 was performed using the Trasis AllinOne (AIO) synthesizer. The process began with the transfer and trapping of approximately 37 GBq of [^18^F]fluoride on a QMA cartridge. The reactor was initially heated to 65 °C. Subsequently, a syringe was prepared containing 2 mg of tetraethylammonium bicarbonate in 1 mL of methanol along with 0.8 mL of air. This mixture was then used to elute the [^18^F]fluoride into the reactor. The reactor temperature was increased to 80 °C, followed by a drying phase consisting of three steps under vacuum with nitrogen: 80 s at 115 °C, 180 s at 125 °C, and 124 s at 95 °C. After drying, the reactor was cooled to 55 °C, and 5 mg of precursor along with 20 mg of tetrakis(pyridine)copper(II)triflate in 0.8 mL of *N*,*N*-dimethylacetamide (DMA) were added. The ^18^F-fluorination reaction proceeded at 110 °C for 20 min. Post-reaction, the reactor was cooled to 40 °C. The resulting mixture was purified by reversed-phase high-performance liquid chromatography (RP-HPLC) using a Phenomenex Luna 5μ C18 column (100 Å, 250 x 10 mm). The mobile phase consisted of an acetonitrile/water mixture (1:3) with 5.4 g of ammonium acetate (NH_4_Ac) at pH 4.4. Further purification was achieved through solid-phase extraction (SPE) using a C18 cartridge, with elution performed using ethanol and washing with a 0.9% saline solution. The preparation of [^18^F]SynVesT-1 resulted in a radiochemical yield (rcy) of up to 20% and a radiochemical purity of 99%. The radiotracer was diluted with sterile saline solution (0.9%) and injected as intravenous bolus (10 ml in 0.5 min). The mean injected dose of [^18^F]SynVesT-1 was 190.9 ± 33.3 MBq, mean molar activity at injection time was 32.4 ± 32.8 GBq/µmol. Injection and scan were started simultaneously at 11:35:23 ± 01:00:24 on average and scan duration was 90 min. Time period between wake up and scan start was 4.2 ± 0.7 h on average (28.0 ± 2.0 h for the sleep deprivation condition).

[^18^F]SynVesT-1 PET data acquisition and magnetic resonance (MR) were conducted on a 3 Tesla PET/MR system (BrainPET; Siemens Healthineers). Participants’ constant wakefulness during PET scans was checked by video monitoring of subjects’ eye blink behavior. PET data were acquired in list-mode and reconstructed into 3D images with 256 x 256 x 153 voxels of size 1.25^3^ mm^3^ using the OP-OSEM reconstruction algorithm with 2 iterations and 32 subsets. The list-mode data was split into 34 consecutive time frames with increasing lengths, i.e., 6 x 10 s, 3 x 20 s, 3 x 30 s, 4 x 60 s, 3 x 150 s, 15 x 300 s. Detection sensitivity normalization, decay correction, dead time correction, random correction, scatter correction (Single Scatter Simulation with scaling by tail fitting), attenuation correction, and 2.5 mm Gaussian post-filtering was applied to each of the 34 image frames. For the attenuation and scatter correction, a combined attenuation map obtained from a transmission scan of the transmit/receive head coil and a MR-image derived patient attenuation map was used [49].

### Data analysis

Preprocessing of PET and MR data was done with the PMOD Neuro Tool pipeline (v 4.006; PMOD Technologies): dynamic image frames were realigned to an average of frames 1–14 to correct for potential head movements and co-registered with the MRI, which was segmented into gray and white matter and cerebrospinal fluid compartments, and normalized to the MNI (Montreal Neurological Institute) space. The maximal head movement (translation and rotation from realignment) occurring during each scan was not significantly different between scan one and two, neither in the control group nor the sleep deprivation group and was subsequently not entered as a confounding variable into the analysis. Volumes of interest (VOIs) were defined by the intersection of the automated anatomical labeling (AAL) template in the MNI space implemented in the PMOD software [50] and the gray matter segments from the individual MRI. For quantification the SRTM2 [51] with a to 2 ml in volume eroded centrum semiovale reference region and a population-based fixed k2‘of 0.032 was used [40]. Side averaged volumes of interest were included in the analysis: frontal, cingulate, occipital, parietal and temporal cortex, hippocampus, thalamus, and striatum (and insula only for correlation in Fig 4). For visualization purposes, parametric BP_ND_ images were generated with voxelwise SRTM2 and surface mapping with CAT12 [52].

### Performance and sleepiness

A 3-min version of the PVT was used to measure vigilant attention. [53]. Reaction times >500 ms were defined as lapses. Mean reaction speed was analyzed by calculating the relative difference to the corresponding baseline value of the first PET scan. For the number of lapses, the absolute difference to the corresponding baseline value was calculated, as most of the baseline values were zero. Sleepiness was measured on the KSS [54].

### Sleep EEG recordings

Sleep was recorded using a wireless headband (DREEM 3 headband, Rhythm, Paris, France; now Beacon Biosignals, Inc, Boston, United States) [55]. The EEG headband is equipped with five dry EEG electrodes (occipital: O1, O2, frontal: Fp1, F7, and F8 sampled at 250 Hz), a 3D accelerometer for measuring movement, head position, and respiratory rate.

### EEG analysis

The analysis of SWA (relative delta EEG power in the 0.5 to 4 Hz range, using a Welch periodogram with a 4-s Hamming window, command: yasa.bandpower(data)) during the first 90 min (first NREM/REM sleep cycle) was conducted using the python based YASA toolbox (Version 0.6.3) [56]. In two subjects, either the baseline or recovery sleep EEG could not be analyzed due to bad signal quality.

### Statistical analysis

All analyses were conducted using SAS version 9.4. In all statistical tests, the significance level was set at *p* < 0.05. Normal distribution of residuals was verified with Q-Q plots and Kolmogorov–Smirnov test. Mixed ANOVAs were calculated on the difference between scan 1 and 2 with group (control and sleep deprivation) and brain region (*n* = 8) and the interaction between group and brain region as fixed factors and subject as random factor. Results were sliced per brain region and *p*-values post-hoc adjusted for multiple comparisons using the false discovery rate. To confirm that data of scan 1 was not different from scan 2 in the control group, a mixed ANOVA was calculated with scan (1 versus 2), brain region (*n* = 8) and interaction between scan and brain region as fixed factors and subject as random factor. Sliced results per brain regions indicated no differences between scans.

Pearson’s product-moment correlation coefficients were calculated to investigate whether there is a linear relationship between the individual change in SWA, cognitive performance (PVT), and sleepiness (KSS) and change in SV2A-indexed synaptic density from baseline to sleep deprivation. The difference between the two PVT and KSS conditions was compared with an unpaired Wilcoxon test.

## Supporting information

S1 Table Study participants’ demographic and experimental parameters. (DOCX)

S2 Table Correlations between the difference (Δ) of regional SV2A indexed synaptic density values (binding potential BP_ND_) and slow wave activity (SWA) after sleep deprivation and baseline. The data underlying this Table are available here: S3 Data.
(DOCX)

S3 Table Effect of sleep deprivation on cognition. (DOCX)

S1 Data Individual values corresponding to Table 1 and Fig 3: Regional SV2A-indexed synaptic density values (binding potential BPND). (XLSX)

S2 Data Individual values corresponding to S1 Table: study participants’ demographic and experimental parameters. (XLSX)

S3 Data Individual values corresponding to S2 Table: Difference (Δ) of regional SV2A-indexed synaptic density values (binding potential BP_ND_) and slow wave activity (SWA) after sleep deprivation and baseline. (XLSX)

S4 Data Individual values corresponding to S3 Table: Effect of sleep deprivation on cognition (Karolinska Sleepiness Scale, Psychomotor vigilance task (PVT): mean reaction speed (1/s) and slowest 10% of reaction speed (1/s). (XLSX)

## Abbreviations

AAL: automated anatomical labeling
DMA: *N,N*-dimethylacetamide
ICCs: intraclass correlation coefficients
KSS: Karolinska Sleepiness Scale
mGluR5: metabotropic glutamate receptor
MR: magnetic resonance
MRS: magnetic resonance spectroscopy
PET: positron emission tomography
PVT: psychomotor vigilance task
RP-HPLC: reversed-phase high-performance liquid chromatography
SHY: synaptic homeostasis hypothesis
SPE: solid-phase extraction
SRTM2: simplified reference tissue model 2
SV2A: synaptic vesicle glycoprotein 2A
SWA: slow wave activity
TIB: time-in-bed
TMS: transcranial magnetic stimulation
VOIs: volumes of interest.
