# Supplementary material for: Sleep deprivation increases levels of the synaptic density marker SV2A in the human brain
Source: PLoS Biol. 2026 Jun 23;24(6):e3003816. doi: 10.1371/journal.pbio.3003816 (PMC13289872; doi:10.1371/journal.pbio.3003816)
Supplement: S1 Table — (DOCX) [file pbio.3003816.s001.docx]

S1 Table.

Study participants’ demographic and experimental parameters.

|  |  | Control group | Sleep deprivation group | p-value^#^ |
| --- | --- | --- | --- | --- |
| N |  | 20 (7f) | 20 (7f) | n.a. |
| Age (years) |  | 29.8 ± 7.4 | 26.0 ± 4.8 | 0.06 |
| BMI (kg/m²) |  | 24.9 ± 3.0 | 25.0 ± 3.9 | 0.98 |
| Clock time of scanning |  | 11:37 AM | 11:34 AM | 0.79 |
| Injected radioactivity (MBq) | Scan 1  Scan 2  p-value^§^ | 188.9 ± 28.7  189.6 ± 26.6  0.57 | 190.1 ± 37.1  195.1 ± 41  0.16 |  |
| Injected mass (nmol) | Scan 1  Scan 2  p-value^§^ | 8.7 ± 5.5  7.3 ± 4  0.23 | 10.5 ± 6.6  11.1 ± 9.3  0.69 |  |
| Time awake | Scan 1  Scan 2  p-value^§^ | 4.3 ± 0.8  4 ± 0.6  0.03 | 4.4 ± 0.6  28 ± 0.7  ^<^0.01 |  |
| Becks Depression Inventory II | Scan 1  Scan 2  p-value^§^ | 3.1 ± 4.9  1.6 ± 1.2  0.2 | 2.6 ± 3.8  3.1 ± 3.3  0.6 |  |

Values are given as mean ± standard deviation. ^#^Unpaired t-test (groups)/^§^paired t-test (scans). Abbreviation: BMI = body mass index.
