# Supplementary material for: Sleep deprivation increases levels of the synaptic density marker SV2A in the human brain
Source: PLoS Biol. 2026 Jun 23;24(6):e3003816. doi: 10.1371/journal.pbio.3003816 (PMC13289872; doi:10.1371/journal.pbio.3003816)
Supplement: S2 Table — The data underlying this Table are available here: S3 Data. (DOCX) [file pbio.3003816.s002.docx]

S2 Table. Correlations between the difference (Δ) of regional synaptic density values (binding potential BP_ND_) and slow wave activity (SWA) after sleep deprivation and baseline

| Δ BP_ND_ |  | Δ SWA |  | Location of electrodes | | | | |
| --- | --- | --- | --- | --- | --- | --- | --- | --- |
|  |  |  |  | F7-F8 |  | Average F7-F8, F7-O1 F8-O2 | | |
| Frontal ctx |  | Pearson's r |  | 0.45 |  | 0.473 | * |  |
|  |  | p-value |  | 0.061 |  | 0.047 |  |  |
| Striatum |  | Pearson's r |  | 0.587 | * | 0.578 | * |  |
|  |  | p-value |  | 0.01 |  | 0.012 |  |  |
| Thalamus |  | Pearson's r |  | 0.579 | * | 0.602 | ** |  |
|  |  | p-value |  | 0.012 |  | 0.008 |  |  |
| Cingulate ctx |  | Pearson's r |  | 0.396 |  | 0.432 |  |  |
|  |  | p-value |  | 0.104 |  | 0.073 |  |  |
| Occipital ctx |  | Pearson's r |  | 0.419 |  | 0.509 | * |  |
|  |  | p-value |  | 0.084 |  | 0.031 |  |  |
| Temporal ctx |  | Pearson's r |  | 0.356 |  | 0.43 |  |  |
|  |  | p-value |  | 0.147 |  | 0.075 |  |  |
| Parietal ctx |  | Pearson's r |  | 0.548 | * | 0.601 | ** |  |
|  |  | p-value |  | 0.019 |  | 0.008 |  |  |
| Hippocampus |  | Pearson's r |  | 0.373 |  | 0.434 |  |  |
|  |  | p-value |  | 0.127 |  | 0.072 |  |  |
| Average cortical region | | Pearson's r |  | 0.473 | * | 0.537 | * |  |
|  |  | p-value |  | 0.047 |  | 0.021 |  |  |
| Avg. of insula cing. ctx. | | Pearson's r |  | 0.528 | * | 0.563 | * |  |
|  |  | p-value |  | 0.024 |  | 0.015 |  |  |
| * p < .05, ** p < .01 | | |  |  |  |  |  |  |
