# Supplementary material for: Sleep deprivation increases levels of the synaptic density marker SV2A in the human brain
Source: PLoS Biol. 2026 Jun 23;24(6):e3003816. doi: 10.1371/journal.pbio.3003816 (PMC13289872; doi:10.1371/journal.pbio.3003816)
Supplement: S3 Table — (DOCX) [file pbio.3003816.s003.docx]

S3 Table. Effect of sleep deprivation on cognition

| **Measurement** | **Baseline** | **Sleep deprivation** | ***p*** |
| --- | --- | --- | --- |
| Karolinska Sleepiness Scale | 2.9 ± 1.4 | 6.2 ± 1.8 | <0.0001 |
| PVT |  |  |  |
| Mean reaction speed (1/s) | 4.4 ± 0.5 | 4.1 ± 0.5 | 0.0032 |
| Slowest 10% of reaction speed (1/s) | 3.5 ± 0.4 | 3.2 ± 0.4 | 0.0085 |

p: Unpaired Wilcoxon comparing differences to baseline between both groups
mean±SD
