# Supplementary material for: Sleep deprivation increases levels of the synaptic density marker SV2A in the human brain
Source: PLoS Biol. 2026 Jun 23;24(6):e3003816. doi: 10.1371/journal.pbio.3003816 (PMC13289872; doi:10.1371/journal.pbio.3003816)
Supplement: Attachment — Submitted filename: Response_2.docx [file pbio.3003816.s010.docx]

**Response to Reviewers**

We would like to thank the PLOS Biology editors, the academic editor and the reviewers for providing very positive and encouraging comments and the willingness to evaluate our revisions. We also thank the reviewers for the suggestions that significantly improved the manuscript. We have made a point-by-point response or revision corresponding to the comments. For clarity, our responses are given after each of the reviewer’ comments (the comments are italics followed by our responses). We also systematically reported the manuscript changes in our point-by-point response.

**reviewer #1**

*1. This interesting study reports changes in SV2A expression in humans after a night of sleep or a night of sleep deprivation. While no significant changes were observed in controls allowed to sleep, small increases in SV2A expression were seen in 6 of the 8 brain regions tested after sleep deprivation, including several cortical areas, hippocampus and striatum. The authors frame this result as supporting the synaptic homeostasis hypothesis (although the first 2 references do not seem the most relevant for this hypothesis). The results are straightforward and the study is well conducted. Some clarifications, however, are needed, especially in the interpretation.*

Thank you very much for this supportive comment. We added two more early references regarding SHY:

Tononi G, Cirelli C. Sleep function and synaptic homeostasis. Sleep Med Rev. 2006;10(1):49–62.

Tononi G, Cirelli C. Sleep and synaptic homeostasis: a hypothesis. Brain Res Bull. 2003;62(2):143–50.

*2. First, the authors suggest only late in the discussion that caution is needed to interpret SV2A results, but this caution is not taken apparent in the title or in most of the main text of the paper (see description of the 2 main goals of the study at the end of the introduction), where it is assumed that indeed changes in SV2A reflect changes in "synaptic terminal density" (bouton number?). My understanding is that it remains unclear whether SV2A levels reflect the number of axonal boutons and/or the status of the synaptic machinery in the pre-synapse (more SV2A molecules /vesicle, more synaptic vesicles?). Since SV2A is expressed in both excitatory and inhibitory synapses, changes after sleep deprivation could also reflect a switch in the excitatory/inhibitory tone in the brain after sleep deprivation.*

We thank the reviewer for this important and thoughtful comment regarding the interpretation and consistency of SV2A PET measures. In response, we have revised the manuscript to ensure a more cautious and consistent use of terminology throughout, and to better reflect the biological ambiguity of the SV2A signal.

First, we have updated the title to: “Sleep deprivation increases SV2A availability as proxy measure of synaptic density in human brains” This change avoids directly equating SV2A PET signal with synaptic density and instead emphasizes SV2A availability as the primary measured outcome.

Second, we have clarified the interpretation of the SV2A signal early in the Introduction by adding the following text: “However, whether this reflects presynaptic terminal number, vesicle complement, SV2A expression per vesicle or excitatory/inhibitory synapse composition cannot be resolved with in vivo imaging. While SV2A availability is commonly interpreted as proxy measure of synaptic density we refer to it here as ‘SV2A-indexed synaptic density’ to reflect this interpretation while acknowledging its underlying biological ambiguity.” This addition explicitly acknowledges the multiple biological contributors to the PET signal and defines our terminology upfront.

Third, we have expanded the Discussion to address the lack of specificity with respect to synapse type by adding: “Given that SV2A is present in both excitatory and inhibitory synapses, the observed fluctuations in tracer binding may reflect a net change in the brain's excitatory/inhibitory (E/I) tone following sleep loss. The inherent lack of specificity for a particular neurotransmitter system remains a limitation in interpreting whether these results represent a targeted or global synaptic response.”

Finally, we have ensured consistent terminology across the manuscript by using “SV2A availability” as the primary term, and “SV2A-indexed synaptic density” only where interpretative context is intended.

We believe these revisions address the reviewer’s concerns by clearly distinguishing between what is measured (SV2A availability) and how it may be interpreted, while explicitly acknowledging the current limitations of in vivo SV2A PET imaging.

*3. I encourage the authors to rephrase the statement (line 211) that SV2A changes after sleep deprivation have not been investigated in animal models. If recent SV2A results in mice after sleep and sleep deprivation are published soon, there will be a need to understand possible discrepancies between results in rodents and humans. These authors seem to be in the best position to address these differences.*

We thank the reviewer for the opportunity to comment on our recent efforts to investigate SV2A changes after sleep deprivation in mice. We fully agree that comparing human and rodent findings will be important for interpreting potential species‑specific differences. Indeed, we have performed extensive rodent experiments. The multi modal approach comprising in‑vivo PET imaging, autoradiography, Golgi spine quantification and electron microscopy—are part of an ongoing project, and the data are still being analyzed and prepared for publication. Given the methodological complexity and the need to present these results with appropriate detail and validation, we prefer not to include preliminary or unpublished findings in this manuscript. We plan to report and discuss these data comprehensively in a separate manuscript. We removed the sentence that changes have not been investigated in animal models.

*Minor points:*

*4. Line 172: this comment is unclear; do the authors suggest that SD subjects could sleep to some extent before the nap? or that "renormalization" happens during waking? A similar comment appears later in the text.*

We realize that the word “renormalization” might be misleading. To clarify: the subjects had no nap opportunity. The sentence was rephrased to: “These relatively small but reliable effects are notable given the temporal offset (~4 hours post-wake) before baseline PET scanning, which may have allowed partial early wake‑dependent changes of synaptic components to develop, diminishing the magnitude of the difference compared with the sleep‑deprived condition.”

*5. Line 191: this sentence needs to be fixed*

The sentence was adopted: “Slow wave sleep drives reduction of synaptic AMPA receptor levels in rat neocortex and hypothalamus (21).”

**reviewer #2**

*Reviewer #2: The presented research tests the Synaptic Homeostasis Hypothesis (SHY), which posits that wakefulness is associated with a net increase in synaptic strength driven by learning-related plasticity, whereas sleep facilitates synaptic renormalization (or down-selection) to maintain network efficiency and cellular homeostasis. To address this hypothesis, the authors quantify changes in synaptic density following 28 hours of total sleep deprivation using positron emission tomography (PET) imaging.*

*SHY is strongly supported by animal studies; however, direct evidence in humans remains limited. The use of advanced PET imaging to assess synaptic density therefore represents a valuable and innovative contribution to the field and provides important translational relevance to one of the leading hypotheses concerning sleep function. The study design is well conceived, the analytical approach is appropriate and transparent, and the manuscript is clearly written.*

Thank you very much for this supportive comment.

*The reviewer has only minor concerns:*

*1.	Abstract: It would be helpful to specify when the PET scans were performed (e.g., before or after sleep or sleep deprivation) to improve clarity for the reader.*

To clarify we added the following: “Half of the participants were assigned to the normal‑sleep (i.e. control) condition and half to the sleep‑deprivation condition. Scans were performed at the same circadian time point, approximately 4 hours after awakening in the control group and during baseline in the sleep-deprivation group or after ~28 hours of continuous wakefulness in the sleep‑deprivation group after sleep deprivation.”

*2.	Terminology related to SHY: Earlier publications introduced the term "downscaling," whereas more recent work, including some cited by the authors, uses "renormalization" or "down-selection." The terminology should be updated and applied consistently throughout the manuscript.*

In line 77 “downscaling” and in the abstract “renormalization” was replaced by “down-selection”.

*3.	Referencing of SHY: It is unclear why a commentary (Ref. 2) is cited instead of the original publication in which SHY was first proposed. The primary source should be referenced.*

We added two earlier references regarding SHY.

Tononi G, Cirelli C. Sleep function and synaptic homeostasis. Sleep Med Rev. 2006;10(1):49–62.

Tononi G, Cirelli C. Sleep and synaptic homeostasis: a hypothesis. Brain Res Bull. 2003;62(2):143–50.

*4.	Introduction: The paragraph on depressive disorders appears tangential to the stated study objectives and is not necessary for understanding the rationale. The reviewer suggests removing this section to improve focus.*

We agree and moved that section to the discussion where we had already discussed therapeutic effects of sleep deprivation.

*5.	Figure 1: The terminology used in the text and figure should be harmonized (e.g., "sleep satiation"). In addition, the inclusion of more fine-grained time markers would improve clarity, for example to indicate when the 2-hour EEG recording was conducted. In general, the study design figure is not so easy to understand, for example because the control group is only indicated/included as text. A graphical illustration that includes controls and clearly shows what measurements were compared would be very helpful.*

The terminology has been harmonized and the figure adapted accordingly. (fine grained time markers and separate timelines for control and SD group). Figure 1 is now only showing the study design and the surface projections have been moved to a new figure 2. Therefore, the figure naming of the subsequent ones has been updated.

*6.	Discussion: Direct comparison of effect magnitudes between human and mice studies should be interpreted with caution, as cross-species differences in methodology and scale limit direct comparability.*

The following section was added to the discussion: “However, direct comparison of effect magnitudes across species should be interpreted with caution. Differences in brain architecture, sleep–wake patterns, tracer biology, and the spatial scales captured by PET versus microscopy or biochemical assays limit the extent to which quantitative values can be directly equated. Thus, our comparison is intended to provide qualitative context rather than imply strict numerical equivalence.”

*7.	Contextualization: The discussion of converging evidence could be strengthened by including relevant findings from magnetic resonance spectroscopy studies.*

We added the following paragraph to the discussion: “Our findings align with multimodal evidence from magnetic resonance spectroscopy (MRS) studies showing that sleep deprivation increases neurochemical markers associated with synaptic activity. Converging proton MRS evidence demonstrates that prolonged wakefulness elevates glutamatergic tone—reflected in increased glutamate or GLX levels, higher Glu/GABA ratios, and corresponding increases in mGluR5 availability—and complementary MRS findings from multimodal PET/MRS work further show sleep loss–related changes in metabolites such as myo inositol and glycine, which are linked to astroglial regulation of glutamatergic signaling and sleep pressure (22, 26, 27).”

*8.	Outlook: The future directions section would benefit from a broader perspective. While sleep deprivation may have transient therapeutic effects in depressive disorders, it also has numerous adverse consequences in both healthy and clinical populations. A more balanced outlook would enhance the manuscript's impact.*

We added the following paragraph to the discussion to broaden the outlook: "While acute sleep deprivation may transiently upregulate SV2A-indexed synaptic density, future work should examine the synaptic consequences of repeated or chronic sleep loss, which is associated with cognitive impairment, neuroinflammation, and accelerated neurodegeneration in both healthy and clinical populations. Longitudinal SV2A PET studies spanning recovery sleep and chronic sleep restriction protocols will be essential to determine whether these synaptic changes are fully reversible or carry cumulative neural costs."

**reviewer #3**

*Reviewer #3: In this randomized imaging study in 40 healthy adults, the authors evaluated the relationship between sleep deprivation and synaptic changes. They found significant increases in SV2A PET signal in multiple brain regions following sleep loss. The positive correlation between SV2A radioligand binding and elevated slow wave activity during recovery sleep strengthens the link between synaptic changes and sleep pressure. This study provides meaningful in vivo evidence supporting the synaptic homeostasis hypothesis and highlights SV2A PET imaging as a sensitive method for tracking sleep-wake dependent synaptic changes in humans.*

*This is a well-designed study using a recently developed PET tracer. While SV2A PET has been used to assess synaptic density in various neurological conditions, prior studies specifically investigating sleep or sleep deprivation with SV2A PET have so far not been reported. The manuscript is clear and the discussion is appropriate.*

Thank you very much for this supportive comment.

*1. Throughout the manuscript: there are different SV2A biomarker interpretations: synaptic density (in title), presynaptic terminal density (abstract), and synaptic terminal density (in introduction). Please make this consistent and elaborate on differences in the already established discussion paragraph.*

We thank the reviewer for this important and thoughtful comment regarding the interpretation and consistency of SV2A PET measures. In response, we have revised the manuscript to ensure a more cautious and consistent use of terminology throughout, and to better reflect the biological ambiguity of the SV2A signal.

First, we have updated the title to: “Sleep deprivation increases SV2A availability as proxy measure of synaptic density in human brains” This change avoids directly equating SV2A PET signal with synaptic density and instead emphasizes SV2A availability as the primary measured outcome.

Second, we have clarified the interpretation of the SV2A signal early in the Introduction by adding the following text: “However, whether this reflects presynaptic terminal number, vesicle complement, SV2A expression per vesicle or excitatory/inhibitory synapse composition cannot be resolved with in vivo imaging. While SV2A availability is commonly interpreted as proxy measure of synaptic density we refer to it here as ‘SV2A-indexed synaptic density’ to reflect this interpretation while acknowledging its underlying biological ambiguity.” This addition explicitly acknowledges the multiple biological contributors to the PET signal and defines our terminology upfront.

Third, we have expanded the Discussion to address the lack of specificity with respect to synapse type by adding: “Given that SV2A is present in both excitatory and inhibitory synapses, the observed fluctuations in tracer binding may reflect a net change in the brain's excitatory/inhibitory (E/I) tone following sleep loss. The inherent lack of specificity for a particular neurotransmitter system remains a limitation in interpreting whether these results represent a targeted or global synaptic response.”

Finally, we have ensured consistent terminology across the manuscript by using “SV2A availability” as the primary term, and “SV2A-indexed synaptic density” only where interpretative context is intended.

We believe these revisions address the reviewer’s concerns by clearly distinguishing between what is measured (SV2A availability) and how it may be interpreted, while explicitly acknowledging the current limitations of in vivo SV2A PET imaging.

*2. Throughout manuscript: "Synaptic vesicle protein 2A" should read "Synaptic vesicle glycoprotein 2A"; see UniProt: Q7L0J3.*

“glyco” was added throughout the manuscript.

*3.1 Results: 8 brain regions were evaluated by ROI analysis. Why were these brain regions selected?*

These brain regions were selected based on their susceptibility to the effects of sleep deprivation in previous publications investigating metabotropic glutamate receptors and adenosine receptors.

Holst SC, Sousek A, Hefti K, Saberi-Moghadam S, Buck A, Ametamey SM, et al. Cerebral mGluR5 availability contributes to elevated sleep need and behavioral adjustment after sleep deprivation. Elife. 2017;6.

Weigend S, Holst SC, Treyer V, O'Gorman Tuura RL, Meier J, Ametamey SM, et al. Dynamic changes in cerebral and peripheral markers of glutamatergic signaling across the human sleep-wake cycle. Sleep. 2019;42(11).

Elmenhorst D, Elmenhorst EM, Hennecke E, Kroll T, Matusch A, Aeschbach D, et al. Recovery sleep after extended wakefulness restores elevated A1 adenosine receptor availability in the human brain. ProcNatlAcadSciUSA. 2017;114(16):4243–8.

3.2 *Was a global effect expected?*

A global effect was not necessarily expected, as prior human MRI and PET studies of sleep–wake manipulations have typically reported regionally heterogeneous changes rather than uniform whole‑brain shifts. While sleep deprivation is thought to influence widespread synaptic physiology, the magnitude and spatial distribution of SV2A‑indexed changes remain uncertain, making both regional and global patterns plausible but not guaranteed.

*3.3 Did the authors consider an exploratory SPM analysis to evaluate potential changes beyond these regions?*

We considered an exploratory voxelwise SPM analysis, but a pre‑analysis power calculation indicated that such an approach would be underpowered with 20 participants per condition. Even assuming moderate‑to‑large single‑voxel effect sizes, the expected uncorrected power remained limited, and whole‑brain family‑wise error (FWE) correction across approximately 50,000 voxels would make surviving clusters highly unlikely. Additionally, the voxelwise pharmacokinetic modelling is inherently more variable compared to the atlas approach due to the higher noise in the single voxel time activity curves in comparison to those of much larger volumes of interest. Nonetheless, we now conducted three complementary voxelwise analyses (t‑tests on scan‑to‑scan differences, ANCOVA, and mixed ANOVA) using two smoothing kernels (8 mm and 12 mm). As anticipated, no clusters survived whole‑brain FWE correction in any of these analyses.

*4. Results: could reduced motion in scan 2 in the sleep-deprived subjects have contributed to the study outcome? How did the authors control for a potential contribution? Were subjects allowed to sleep during PET?*

Thank you for reminding us to include the important information regarding sleep inside the scanner (which was not allowed and wakefulness was monitored continuously): “Participants’ constant wakefulness during PET scans was checked by video monitoring of subjects’ eye blink behavior.” was added to the methods section.

Head movement inside the scanner was minimized by individually stabilizing the subjects head with several memory foam cushions. As implemented in the PET analysis software Pmod’s neurotool pipeline, we realigned each reconstructed PET image of the dynamic series to an average image of the first minutes of each scan (frames 1-14) during preprocessing. This information is described now in more detail in the methods.

“Preprocessing of PET and MR data was done with the PMOD Neuro Tool pipeline (v 4.006; PMOD Technologies): Dynamic image frames were realigned to an average of frames 1-14 to correct for potential head movements and co-registrered with the MRI which was segmented into grey and white matter and cerebrospinal fluid compartments, and normalized to the MNI (Montreal Neurological Institute) space. Volumes of interest (VOIs) were defined by the intersection of the automated anatomical labelling (AAL) template in the MNI space implemented in the PMOD software and the grey matter segments from the individual MRI.”

“The maximal head movement (translation and rotation from realignment) occurring during each scan was not significantly different between scan one and two neither in the control group nor the sleep deprivation group and was subsequently not included as a confounding variable into the analysis.”

*5. S2 Table. Clarify abbreviation dSWA.*

The explanation of the abbreviation was included in the table caption. (“Correlations between the difference (Δ) of regional synaptic density values (binding potential BPND) and slow wave activity (SWA) after sleep deprivation and baseline”)

*6. Figure 1: the study design figure is confusing. Can you indicate which subject groups underwent the events in each row? Is the time indication relevant for all rows?*

We have adopted the figure to make the timeline and groups more clear.

*7. Figure 3: which brain regions were used for the SV2A BPnd values presented in* *bottom graphs?*

We had originally mentioned the brain regions in the title of the scatter plots. To clarify we now moved the region specification into the respective axis caption.

*8. Discussion, line 208 - please confirm that this was five SV2A molecules or five SV2 molecules in the reference.*

Thank you for pointing out this typo. The reference reported the number of SV2 molecules. This was corrected in the manuscript.

*9. Discussion: The authors did not discuss in detail the literature on potential synaptophysin changes in preclinical studies. Since this protein is closely associated with SV2A, it would be beneficial to elaborate on this literature.*

“SV2A binding has been shown to correlate with synaptophysin in postmortem tissue (20-22), an integral synaptic vesicle membrane protein that interacts with the SNARE complex and regulates vesicle formation and neurotransmitter release. Studies in male rodents have shown no change or more often decreases in synaptophysin after 24 to 96 hours of sleep deprivation using cage shaking or mainly the multiple platform over water method where animals will fall into the water during REM sleep associated muscle atonia (1-4) (reviewed in (5)), However, the translation of results from rodents to humans is limited due to the methodological differences used to enforce wakefulness. Most rodent experiments rely on long and often stressful (life-threatening) deprivation protocols which induce physiological stress responses that are difficult to separate from sleep loss effects.”

***Journal Requirements:***

*1) Please ensure your short title is correct in the submission form, including symbols, italicisation etc. Your short title will be typeset from the submission form directly.*

done

*2) Please ensure that the funders and grant numbers match between the Financial Disclosure field and the Funding Information tab in your submission form. Note that the funders must be provided in the same order in both places as well.*

*Currently, "FWO and FRQS" are missing from the Funding Information*

There is only one grant number for all three cooperating funding organisations.

*3) Please provide separate figure files in .tif or .eps format. For more information about figure files please see our guidelines:*

<https://journals.plos.org/plosbiology/s/figures>

<https://journals.plos.org/plosbiology/s/figures#loc-file-requirements>

done

*4) We have noticed that you have uploaded Supporting Information files, but you have not included a list of legends. Please add a full list of legends for your Supporting Information files after the references list.*

added

*5) Please note that the contact for accessing your data cannot be an author on the manuscript. Please therefore replace the current contact information with a third party contact, such as an institutional data access committee.*

Has been replaced.*6) "Please refer to the following article for PLOS Biology's best-practice recommendations for data presentation:* [*https://journals.plos.org/plosbiology/article?id=10.1371/journal.pbio.1002128*](https://journals.plos.org/plosbiology/article?id=10.1371/journal.pbio.1002128)*.*

*Guidelines for statistical reporting, if relevant, may be found here:* [*https://journals.plos.org/plosbiology/s/best-practices-in-research-reporting#loc-statistical-reporting*](https://journals.plos.org/plosbiology/s/best-practices-in-research-reporting#loc-statistical-reporting)."

done

*7) Some material included in your submission may be copyrighted. According to PLOS’s copyright policy, authors who use figures or other material (e.g., graphics, clipart, maps) from another author or copyright holder must demonstrate or obtain permission to publish this material under the Creative Commons Attribution 4.0 International (CC BY 4.0) License used by PLOS journals. Please closely review the details of PLOS’s copyright requirements here: PLOS Licenses and Copyright. If you need to request permissions from a copyright holder, you may use PLOS's Copyright Content Permission form.*

*Please respond directly to this email and provide any known details concerning your material's license terms and permissions required for reuse, even if you have not yet obtained copyright permissions or are unsure of your material's copyright compatibility. Once you have responded and addressed all other outstanding technical requirements, you may resubmit your manuscript within Editorial Manager.*

*Potential Copyright Issues: [Figure 1]: Please confirm whether you drew the images / clip-art within the figure panels by hand. If you did not draw the images, please provide (a) a link to the source of the images or icons and their license / terms of use; or (b) written permission from the copyright holder to publish the images or icons under our CC BY 4.0 license. Alternatively, you may replace the images with open source alternatives. See these open source resources you may use to replace images / clip-art: (-* [*https://commons.wikimedia.org*](https://commons.wikimedia.org)*,-* [*https://openclipart.org/*](https://openclipart.org/)*)*

I confirm that I *drew the images.*
