# Supplementary material for: Sleep deprivation increases levels of the synaptic density marker SV2A in the human brain
Source: PLoS Biol. 2026 Jun 23;24(6):e3003816. doi: 10.1371/journal.pbio.3003816 (PMC13289872; doi:10.1371/journal.pbio.3003816)
Supplement: Attachment — Submitted filename: Response_to_R2.docx [file pbio.3003816.s011.docx]

**Response to Revision R2**

We would like to thank you, the PLOS Biology editors and the academic editor for the continuous support and the suggestions.

@1 TITLE: *We like the new title and follow your suggestion. Thank you.*

@2: CLINICAL TRIAL: *We would like to clarify that this study was not a clinical trial. We prospectively register our study protocols in humans regularly with the German Clinical Trials Register to provide transparency regarding objectives and methodology.*

*In the manuscript we added this information: “The study was prospectively registered 19.01.2022 with the German Clinical Trials Registry to provide transparency regarding objectives and methodology (DRKS # DRKS00027867,* [*https://drks.de/search/en/trial/DRKS00027867*](https://drks.de/search/en/trial/DRKS00027867)*) although it was not a clinical trial.”*

@3: DATA

a. Data: We added now the study specific DOI: https://doi.org/10.26165/JUELICH-DATA/GOTGS5

b. While we understand that you cannot share the raw data, if legally allowed, we would ask that you do share the processed numerical data used to generate your figures. This can be provided as a supplementary files (e.g., excel). Please ensure that all data files are uploaded as 'Supporting Information' and are invariably referred to (in the manuscript, figure legends, and the Description field when uploading your files) using the following format verbatim: S1 Data, S2 Data, etc. Multiple panels of a single or even several figures can be included as multiple sheets in one excel file that is saved using exactly the following convention: S1_Data.xlsx (using an underscore).

*- Data files have been renamed*

*-Connected Data Files have been mentioned in the Figure/Table Legends.*

NOTE: the numerical data provided should include all replicates AND the way in which the plotted mean and errors were derived (it should not present only the mean/average values).

*-There were no replicates that could be reported. If standard deviation of standard error of the mean was used is indicated.*

-Please also ensure that figure legends in your manuscript include information on where the underlying data can be found, and ensure your supplemental data file/s has a legend.

*-In the figure legend is now stated which Data file is associated. Supplemental data files have now a legend.*

-Please ensure that your Data Statement in the submission system accurately describes where your data can be found.

*-We added the data availability statement from the manuscripts title page*

(I do see that your paper already has some data files in the supplement - if these are the underlying data for your figures, please make this clearer, by adding a legend for these files, and referencing them in the relevant figure legends).

-*done*

@4: CODE: 4 - CODE: Per journal policy, if you have generated any custom code during the course of this investigation, please make it available without restrictions. Please ensure that the code is sufficiently well documented and reusable, and that your Data Statement in the Editorial Manager submission system accurately describes where your code can be found. More information on our Code Policy, what and how to share can be found here: <https://journals.plos.org/plosbiology/s/code-availability>

*-The code for the main statistical analysis is available at Zenodo. “The codes of the main statistical data analysis are available on Zenodo (https://doi.org/10.5281/zenodo.20033920).”*

5 - EDITORIAL SUGGESTION: We would suggest that you give the manuscript one final pass to ensure wording/terminology consistency, and with an eye for grammatical issues. When reading the revision, we noticed a few places that would benefit from a final edit. For example:

*-manuscript was checked for grammar and typos.*

a. SV2A terminology is better now, but it is still a bit uneven. In most places the revised version describes SV2A availability more cautiously as a proxy for synaptic density, but in a few places it still reads more like a direct measure of presynaptic terminal density. It would be good to do one more pass to make that wording consistent throughout

*-Throughout the manuscript the terminology has been adapted.*

b. The academic editor noticed that there seems to be a small inconsistency in the number of ROIs. In the Results they refer to eight brain regions, but in the Methods the listed ROIs seem to add up to nine. Please double check that and clarify.

- For the exploration of the changes between the build-up of sleep propensity and the increase of SV2A-indexed synaptic density (in Figure 4, lower left panel), we reported additionally to the eight regions included in the mixed ANOVA the generator region of slow wave activity (insula) as suggested by Murphy et al. 2009. To indicate that the insula region was only used in this correlation we added to methods: “(and insula only for correlation in Fig. 4)”

As you address these items, please take this last chance to review your reference list to ensure that it is complete and correct. If you have cited papers that have been retracted, please include the rationale for doing so in the manuscript text, or remove these references and replace them with relevant current references. Any changes to the reference list should be mentioned in the cover letter that accompanies your revised manuscript.

-no changes have been made to the reference list.
