# Supplementary material for: Sleep deprivation increases levels of the synaptic density marker SV2A in the human brain
Source: PLoS Biol. 2026 Jun 23;24(6):e3003816. doi: 10.1371/journal.pbio.3003816 (PMC13289872; doi:10.1371/journal.pbio.3003816)
Supplement: Peer review history

# Editor report: Decision Letter 0

**DOI:** 10.1371/journal.pbio.3003816.r001

**Authors:** Lucas Smith

29 Jan 2026

Dear Dr Elmenhorst,

Thank you for submitting your manuscript entitled "Sleep deprivation increases synaptic density in human brains" for consideration as a Research Article by PLOS Biology.

Your manuscript has now been evaluated by the PLOS Biology editorial staff as well as by an academic editor with relevant expertise and I am writing to let you know that we would like to send your submission out for external peer review.

However, before we can send your manuscript to reviewers, we need you to complete your submission by providing the metadata that is required for full assessment. To this end, please login to Editorial Manager where you will find the paper in the 'Submissions Needing Revisions' folder on your homepage. Please click 'Revise Submission' from the Action Links and complete all additional questions in the submission questionnaire.

Once your full submission is complete, your paper will undergo a series of checks in preparation for peer review. After your manuscript has passed the checks it will be sent out for review. To provide the metadata for your submission, please Login to Editorial Manager (https://www.editorialmanager.com/pbiology) within two working days, i.e. by Jan 31 2026 11:59PM.

*Please note that after some discussion, we think your paper would be best suited for consideration as a "Short Report" (https://journals.plos.org/plosbiology/s/what-we-publish#loc-short-reports). We therefore ask that when you provide your metadata, that you also update the article type to short report. This will not require any changes to the manuscript itself.

If your manuscript has been previously peer-reviewed at another journal, PLOS Biology is willing to work with those reviews in order to avoid re-starting the process. Submission of the previous reviews is entirely optional and our ability to use them effectively will depend on the willingness of the previous journal to confirm the content of the reports and share the reviewer identities. Please note that we reserve the right to invite additional reviewers if we consider that additional/independent reviewers are needed, although we aim to avoid this as far as possible. In our experience, working with previous reviews does save time.

If you would like us to consider previous reviewer reports, please edit your cover letter to let us know and include the name of the journal where the work was previously considered and the manuscript ID it was given. In addition, please upload a response to the reviews as a 'Prior Peer Review' file type, which should include the reports in full and a point-by-point reply detailing how you have or plan to address the reviewers' concerns.

During the process of completing your manuscript submission, you will be invited to opt-in to posting your pre-review manuscript as a bioRxiv preprint. Visit http://journals.plos.org/plosbiology/s/preprints for full details. If you consent to posting your current manuscript as a preprint, please upload a single Preprint PDF.

Feel free to email us at plosbiology@plos.org if you have any queries relating to your submission.

Kind regards,

Luke

Lucas Smith, Ph.D.

Senior Editor

PLOS Biology

lsmith@plos.org

# Peer review: Decision Letter 1

**DOI:** 10.1371/journal.pbio.3003816.r002

**Authors:** Lucas Smith

30 Mar 2026

Dear Dr Elmenhorst,

Thank you for your patience while your manuscript "Sleep deprivation increases synaptic density in human brains" went through peer-review at PLOS Biology and I apologize again for the protracted review process. Your manuscript has now been evaluated by the PLOS Biology editors, an Academic Editor with relevant expertise, and by several independent reviewers.

In light of the reviews, which you will find at the end of this email, we are pleased to offer you the opportunity to address the comments from the reviewers in a revision that we anticipate should not take you very long. As you will see, below, the reviewers find your study to be generally interesting and well done, but they have offered a number of suggestions to strengthen the manuscript further. We think their comments will need to be thoroughly addressed before we can consider your study for publication - but we think the reviews can largely be addressed with textual changes.

We will aim to assess your revised manuscript and your response to the reviewers' comments with our Academic Editor aiming to avoid further rounds of peer-review, although we might need to consult with the reviewers, depending on the nature of the revisions.

In addition to these revisions, you may need to complete some formatting changes, which you will receive in a follow up email. A member of our team will be in touch with a set of requests shortly. If you do not receive a separate email within a few days, please assume that checks have been completed, and no additional changes are required.

We expect to receive your revised manuscript within 1 month - however we are happy to extend this deadline if helpful. Please email us (plosbiology@plos.org) if you have any questions or concerns, or would like to request an extension.

At this stage, your manuscript remains formally under active consideration at our journal; please notify us by email if you do not intend to submit a revision so that we withdraw the manuscript.

**IMPORTANT - SUBMITTING YOUR REVISION**

Your revisions should address the specific points made by each reviewer. Please submit the following files along with your revised manuscript:

1. A 'Response to Reviewers' file - this should detail your responses to the editorial requests, present a point-by-point response to all of the reviewers' comments, and indicate the changes made to the manuscript.

*NOTE: In your point-by-point response to the reviewers, please provide the full context of each review. Do not selectively quote paragraphs or sentences to reply to. The entire set of reviewer comments should be present in full and each specific point should be responded to individually.

You should also cite any additional relevant literature that has been published since the original submission and mention any additional citations in your response.

2. In addition to a clean copy of the manuscript, please also upload a 'track-changes' version of your manuscript that specifies the edits made. This should be uploaded as a "Revised Article with Changes Highlighted " file type.

*Resubmission Checklist*

When you are ready to resubmit your revised manuscript, please refer to this resubmission checklist: https://plos.io/Biology_Checklist

To submit a revised version of your manuscript, please go to https://www.editorialmanager.com/pbiology/ and log in as an Author. Click the link labelled 'Submissions Needing Revision' where you will find your submission record.

Please make sure to read the following important policies and guidelines while preparing your revision:

*Published Peer Review*

Please note while forming your response, if your article is accepted, you may have the opportunity to make the peer review history publicly available. The record will include editor decision letters (with reviews) and your responses to reviewer comments. If eligible, we will contact you to opt in or out. Please see here for more details:

https://blogs.plos.org/plos/2019/05/plos-journals-now-open-for-published-peer-review/

*PLOS Data Policy*

Please note that as a condition of publication PLOS' data policy (http://journals.plos.org/plosbiology/s/data-availability) requires that you make available all data used to draw the conclusions arrived at in your manuscript. If you have not already done so, you must include any data used in your manuscript either in appropriate repositories, within the body of the manuscript, or as supporting information (N.B. this includes any numerical values that were used to generate graphs, histograms etc.). For an example see here: http://www.plosbiology.org/article/info%3Adoi%2F10.1371%2Fjournal.pbio.1001908#s5

*Blot and Gel Data Policy*

We require the original, uncropped and minimally adjusted images supporting all blot and gel results reported in an article's figures or Supporting Information files. We will require these files before a manuscript can be accepted so please prepare them now, if you have not already uploaded them. Please carefully read our guidelines for how to prepare and upload this data: https://journals.plos.org/plosbiology/s/figures#loc-blot-and-gel-reporting-requirements

*Protocols deposition*

To enhance the reproducibility of your results, we recommend that if applicable you deposit your laboratory protocols in protocols.io, where a protocol can be assigned its own identifier (DOI) such that it can be cited independently in the future. Additionally, PLOS ONE offers an option for publishing peer-reviewed Lab Protocol articles, which describe protocols hosted on protocols.io. Read more information on sharing protocols at https://plos.org/protocols?utm_medium=editorial-email&utm_source=authorletters&utm_campaign=protocols

Thank you again for your submission to our journal. We hope that our editorial process has been constructive thus far, and we welcome your feedback at any time. Please don't hesitate to contact us if you have any questions or comments.

Sincerely,

Luke

Lucas Smith, Ph.D.

Senior Editor

PLOS Biology

lsmith@plos.org

----------------------------------------------------------------

REVIEWS:

Reviewer #1: This interesting study reports changes in SV2A expression in humans after a night of sleep or a night of sleep deprivation. While no significant changes were observed in controls allowed to sleep, small increases in SV2A expression were seen in 6 of the 8 brain regions tested after sleep deprivation, including several cortical areas, hippocampus and striatum. The authors frame this result as supporting the synaptic homeostasis hypothesis (although the first 2 references do not seem the most relevant for this hypothesis). The results are straightforward and the study is well conducted. Some clarifications, however, are needed, especially in the interpretation.

First, the authors suggest only late in the discussion that caution is needed to interpret SV2A results, but this caution is not taken apparent in the title or in most of the main text of the paper (see description of the 2 main goals of the study at the end of the introduction), where it is assumed that indeed changes in SV2A reflect changes in "synaptic terminal density" (bouton number?). My understanding is that it remains unclear whether SV2A levels reflect the number of axonal boutons and/or the status of the synaptic machinery in the pre-synapse (more SV2A molecules /vesicle, more synaptic vesicles?). Since SV2A is expressed in both excitatory and inhibitory synapses, changes after sleep deprivation could also reflect a switch in the excitatory/inhibitory tone in the brain after sleep deprivation.

I encourage the authors to rephrase the statement (line 211) that SV2A changes after sleep deprivation have not been investigated in animal models. If recent SV2A results in mice after sleep and sleep deprivation are published soon, there will be a need to understand possible discrepancies between results in rodents and humans. These authors seem to be in the best position to address these differences.

Minor points:

Line 172: this comment is unclear; do the authors suggest that SD subjects could sleep to some extent before the nap? or that "renormalization" happens during waking? A similar comment appears later in the text.

Line 191: this sentence needs to be fixed

Reviewer #2: The presented research tests the Synaptic Homeostasis Hypothesis (SHY), which posits that wakefulness is associated with a net increase in synaptic strength driven by learning-related plasticity, whereas sleep facilitates synaptic renormalization (or down-selection) to maintain network efficiency and cellular homeostasis. To address this hypothesis, the authors quantify changes in synaptic density following 28 hours of total sleep deprivation using positron emission tomography (PET) imaging.

SHY is strongly supported by animal studies; however, direct evidence in humans remains limited. The use of advanced PET imaging to assess synaptic density therefore represents a valuable and innovative contribution to the field and provides important translational relevance to one of the leading hypotheses concerning sleep function. The study design is well conceived, the analytical approach is appropriate and transparent, and the manuscript is clearly written.

The reviewer has only minor concerns:

1.Abstract: It would be helpful to specify when the PET scans were performed (e.g., before or after sleep or sleep deprivation) to improve clarity for the reader.

2.Terminology related to SHY: Earlier publications introduced the term "downscaling," whereas more recent work, including some cited by the authors, uses "renormalization" or "down-selection." The terminology should be updated and applied consistently throughout the manuscript.

3.Referencing of SHY: It is unclear why a commentary (Ref. 2) is cited instead of the original publication in which SHY was first proposed. The primary source should be referenced.

4.Introduction: The paragraph on depressive disorders appears tangential to the stated study objectives and is not necessary for understanding the rationale. The reviewer suggests removing this section to improve focus.

5.Figure 1: The terminology used in the text and figure should be harmonized (e.g., "sleep satiation"). In addition, the inclusion of more fine-grained time markers would improve clarity, for example to indicate when the 2-hour EEG recording was conducted. In general, the study design figure is not so easy to understand, for example because the control group is only indicated/included as text. A graphical illustration that includes controls and clearly shows what measurements were compared would be very helpful.

6.Discussion: Direct comparison of effect magnitudes between human and mice studies should be interpreted with caution, as cross-species differences in methodology and scale limit direct comparability.

7.Contextualization: The discussion of converging evidence could be strengthened by including relevant findings from magnetic resonance spectroscopy studies.

8.Outlook: The future directions section would benefit from a broader perspective. While sleep deprivation may have transient therapeutic effects in depressive disorders, it also has numerous adverse consequences in both healthy and clinical populations. A more balanced outlook would enhance the manuscript's impact.

Reviewer #3: In this randomized imaging study in 40 healthy adults, the authors evaluated the relationship between sleep deprivation and synaptic changes. They found significant increases in SV2A PET signal in multiple brain regions following sleep loss. The positive correlation between SV2A radioligand binding and elevated slow wave activity during recovery sleep strengthens the link between synaptic changes and sleep pressure. This study provides meaningful in vivo evidence supporting the synaptic homeostasis hypothesis and highlights SV2A PET imaging as a sensitive method for tracking sleep-wake dependent synaptic changes in humans.

This is a well-designed study using a recently developed PET tracer. While SV2A PET has been used to assess synaptic density in various neurological conditions, prior studies specifically investigating sleep or sleep deprivation with SV2A PET have so far not been reported. The manuscript is clear and the discussion is appropriate.

I have the following comments:

-Throughout the manuscript: there are different SV2A biomarker interpretations: synaptic density (in title), presynaptic terminal density (abstract), and synaptic terminal density (in introduction). Please make this consistent and elaborate on differences in the already established discussion paragraph.

-Throughout manuscript: "Synaptic vesicle protein 2A" should read "Synaptic vesicle glycoprotein 2A"; see UniProt: Q7L0J3.

-Results: 8 brain regions were evaluated by ROI analysis. Why were these brain regions selected? Was a global effect expected? Did the authors consider an exploratory SPM analysis to evaluate potential changes beyond these regions?

-Results: could reduced motion in scan 2 in the sleep-deprived subjects have contributed to the study outcome? How did the authors control for a potential contribution? Were subjects allowed to sleep during PET?

-S2 Table. Clarify abbreviation dSWA.

-Figure 1: the study design figure is confusing. Can you indicate which subject groups underwent the events in each row? Is the time indication relevant for all rows?

-Figure 3: which brain regions were used for the SV2A BPnd values presented in bottom graphs?

-Discussion, line 208 - please confirm that this was five SV2A molecules or five SV2 molecules in the reference.

-Discussion: The authors did not discuss in detail the literature on potential synaptophysin changes in preclinical studies. Since this protein is closely associated with SV2A, it would be beneficial to elaborate on this literature.

# Author comment: Author response to Decision Letter 2

**DOI:** 10.1371/journal.pbio.3003816.r003

22 Apr 2026

Attachment Submitted filename: Response_2.docx

# Editor report: Decision Letter 2

**DOI:** 10.1371/journal.pbio.3003816.r004

**Authors:** Lucas Smith

1 May 2026

Dear Dr Elmenhorst,

Thank you for your patience while we considered your revised manuscript "Sleep deprivation increases SV2A availability as proxy measure of synaptic density in human brains" for publication as a Short Report at PLOS Biology. This revised version of your manuscript has been evaluated by the PLOS Biology editors and the Academic Editor, who is satisfied by the changes made in this revision.

Based on our Academic Editor's assessment of your revision, we are likely to accept this manuscript for publication. However, before we can do so, we need you to address a number of data and policy related requests in a last revision that we anticipate will not take very long. These are detailed below.

**IMPORTANT - Please address the following editorial requests:

1 - TITLE: We understand that you have been asked to tone down the title of your paper by one of the reviewers, and we think the current version is a good step in that direction. However, we would like to propose a more streamlined version (if you agree, and do not think it is too strong).

We suggest:

"'Sleep deprivation increases levels of the synaptic density marker SV2A in the human brain.'"

2 - CLINICAL TRIAL: Given that your study was part of a pre-registered clinical trial, we ask that you:

a. indicate in the abstract that this work was done as part of a clinical trial and include the registry and registration number.

b. provide a CONSORT checklist or relevant reporting guideline (uploaded as supporting information)

c. provide a CONSORT flow diagram (added to Fig 1 would be best)

d. provide the complete Trial protocol (uploaded as supporting information)

e. If relevant, the manuscript file must include an explanation of any deviation from the trial protocol and any information on statistical methods or participants not indicated in the CONSORT documentation

3 - DATA: We understand from your data and code availability statement, that access to the raw data underlying your study is restricted due to ethical or legal restrictions on sharing a sensitive data. This is an allowable data restriction, and thank you for providing the link to the Julich repository where researchers can request access to this data.

a. I saw that page includes a 'contact' button - but I did not see explicit instructions listed on the website for how researchers should request access. Can you expand your Data and Code Availability statement to include a few more details about how researchers can request access to your data?

b. While we understand that you cannot share the raw data, if legally allowed, we would ask that you do share the processed numerical data used to generate your figures. This can be provided as a supplementary files (e.g., excel). Please ensure that all data files are uploaded as 'Supporting Information' and are invariably referred to (in the manuscript, figure legends, and the Description field when uploading your files) using the following format verbatim: S1 Data, S2 Data, etc. Multiple panels of a single or even several figures can be included as multiple sheets in one excel file that is saved using exactly the following convention: S1_Data.xlsx (using an underscore).

NOTE: the numerical data provided should include all replicates AND the way in which the plotted mean and errors were derived (it should not present only the mean/average values).

-Please also ensure that figure legends in your manuscript include information on where the underlying data can be found, and ensure your supplemental data file/s has a legend.

-Please ensure that your Data Statement in the submission system accurately describes where your data can be found.

(I do see that your paper already has some data files in the supplement - if these are the underlying data for your figures, please make this clearer, by adding a legend for these files, and referencing them in the relevant figure legends).

4 - CODE: Per journal policy, if you have generated any custom code during the course of this investigation, please make it available without restrictions. Please ensure that the code is sufficiently well documented and reusable, and that your Data Statement in the Editorial Manager submission system accurately describes where your code can be found. More information on our Code Policy, what and how to share can be found here: https://journals.plos.org/plosbiology/s/code-availability

Please note that we cannot accept sole deposition of code in GitHub, as this could be changed after publication. However, you can archive this version of your publicly available GitHub code to Zenodo. Once you do this, it will generate a DOI number, which you will need to provide in the Data Accessibility Statement (you are welcome to also provide the GitHub access information). See the process for doing this here: https://docs.github.com/en/repositories/archiving-a-github-repository/referencing-and-citing-content

5 - EDITORIAL SUGGESTION: We would suggest that you give the manuscript one final pass to ensure wording/terminology consistency, and with an eye for grammatical issues. When reading the revision, we noticed a few places that would benefit from a final edit. For example:

a. SV2A terminology is better now, but it is still a bit uneven. In most places the revised version describes SV2A availability more cautiously as a proxy for synaptic density, but in a few places it still reads more like a direct measure of presynaptic terminal density. It would be good to do one more pass to make that wording consistent throughout

b. The academic editor noticed that there seems to be a small inconsistency in the number of ROIs. In the Results they refer to eight brain regions, but in the Methods the listed ROIs seem to add up to nine. Please double check that and clarify.

As you address these items, please take this last chance to review your reference list to ensure that it is complete and correct. If you have cited papers that have been retracted, please include the rationale for doing so in the manuscript text, or remove these references and replace them with relevant current references. Any changes to the reference list should be mentioned in the cover letter that accompanies your revised manuscript.

In addition to these revisions, you may need to complete some formatting changes, which you will receive in a follow up email. A member of our team will be in touch with a set of requests shortly. If you do not receive a separate email within a few days, please assume that checks have been completed, and no additional changes are required.

We expect to receive your revised manuscript within two weeks.

To submit your revision, please go to https://www.editorialmanager.com/pbiology/ and log in as an Author. Click the link labelled 'Submissions Needing Revision' to find your submission record. Your revised submission must include the following:

- a cover letter that should detail your responses to any editorial requests, if applicable, and whether changes have been made to the reference list

- a Response to Reviewers file that provides a detailed response to the reviewers' comments (if applicable, if not applicable please do not delete your existing 'Response to Reviewers' file.)

- a track-changes file indicating any changes that you have made to the manuscript.

NOTE: If Supporting Information files are included with your article, note that these are not copyedited and will be published as they are submitted. Please ensure that these files are legible and of high quality (at least 300 dpi) in an easily accessible file format. For this reason, please be aware that any references listed in an SI file will not be indexed. For more information, see our Supporting Information guidelines:

https://journals.plos.org/plosbiology/s/supporting-information

*Published Peer Review History*

Please note that you may have the opportunity to make the peer review history publicly available. The record will include editor decision letters (with reviews) and your responses to reviewer comments. If eligible, we will contact you to opt in or out. Please see here for more details:

https://plos.org/published-peer-review-history/

*Press*

Should you, your institution's press office or the journal office choose to press release your paper, please ensure you have opted out of Early Article Posting on the submission form. We ask that you notify us as soon as possible if you or your institution is planning to press release the article.

*Protocols deposition*

To enhance the reproducibility of your results, we recommend that if applicable you deposit your laboratory protocols in protocols.io, where a protocol can be assigned its own identifier (DOI) such that it can be cited independently in the future. Additionally, PLOS ONE offers an option for publishing peer-reviewed Lab Protocol articles, which describe protocols hosted on protocols.io. Read more information on sharing protocols at https://plos.org/protocols?utm_medium=editorial-email&utm_source=authorletters&utm_campaign=protocols

Please do not hesitate to contact me should you have any questions.

Sincerely,

Luke

Lucas Smith, Ph.D.

Senior Editor

lsmith@plos.org

PLOS Biology

# Author comment: Author response to Decision Letter 3

**DOI:** 10.1371/journal.pbio.3003816.r005

5 May 2026

Attachment Submitted filename: Response_to_R2.docx

# Editor report: Decision Letter 3

**DOI:** 10.1371/journal.pbio.3003816.r006

**Authors:** Lucas Smith

8 May 2026

Dear Dr Elmenhorst,

Thank you for the submission of your revised Short Report "Sleep deprivation increases levels of the synaptic density marker SV2A in the human brain" for publication in PLOS Biology and thank you for addressing our last editorial requests in this revision. On behalf of my colleagues and the Academic Editor, Guang Yang, I am pleased to say that we can in principle accept your manuscript for publication, provided you address any remaining formatting and reporting issues. These will be detailed in an email you should receive within 2-3 business days from our colleagues in the journal operations team; no action is required from you until then. Please note that we will not be able to formally accept your manuscript and schedule it for publication until you have completed any requested changes.

Please take a minute to log into Editorial Manager at http://www.editorialmanager.com/pbiology/, click the "Update My Information" link at the top of the page, and update your user information to ensure an efficient production process.

PRESS

We frequently collaborate with press offices. If your institution or institutions have a press office, please notify them about your upcoming paper at this point, to enable them to help maximize its impact. If the press office is planning to promote your findings, we would be grateful if they could coordinate with biologypress@plos.org. If you have previously opted in to the early version process, we ask that you notify us immediately of any press plans so that we may opt out on your behalf.

We also ask that you take this opportunity to read our Embargo Policy regarding the discussion, promotion and media coverage of work that is yet to be published by PLOS. As your manuscript is not yet published, it is bound by the conditions of our Embargo Policy. Please be aware that this policy is in place both to ensure that any press coverage of your article is fully substantiated and to provide a direct link between such coverage and the published work. For full details of our Embargo Policy, please visit http://www.plos.org/about/media-inquiries/embargo-policy/.

Thank you again for choosing PLOS Biology for publication and supporting Open Access publishing. We look forward to publishing your study.

Sincerely,

Luke

Lucas Smith, Ph.D.

Senior Editor

PLOS Biology

lsmith@plos.org
